# Severe reverse Takotsubo cardiomyopathy and cardiogenic shock during Caesarean section surgery under epidural anaesthesia: a case report

**DOI:** 10.1093/ehjcr/ytaf619

**Published:** 2025-12-01

**Authors:** Ammar M Alkadi, Fahmi A Alkaf, Muhammad Azam Shah, Mohammed Bara Qattea

**Affiliations:** King Fahad Medical City, Dabab Street, Sulaymaniyah, PO Box 59046, Riyadh 11525, Saudi Arabia; King Fahad Medical City, Dabab Street, Sulaymaniyah, PO Box 59046, Riyadh 11525, Saudi Arabia; King Fahad Medical City, Dabab Street, Sulaymaniyah, PO Box 59046, Riyadh 11525, Saudi Arabia; King Fahad Medical City, Dabab Street, Sulaymaniyah, PO Box 59046, Riyadh 11525, Saudi Arabia

**Keywords:** Case report, Stress cardiomyopathy, Reverse Takotsubo, Peripartum cardiomyopathy, Cardiogenic shock, Heart failure

## Abstract

**Background:**

Takotsubo cardiomyopathy is a type of acute heart failure typically following an emotional or physical stressor. Reverse Takotsubo is a rare pattern representing only 2% of the disease. There are many reported medical triggers, including surgical procedures, intubation, and induction of general anaesthesia.

**Case summary:**

A 27-year-old medically free pregnant woman who developed a sudden cardiogenic shock during caesarean section surgery under epidural anaesthesia. The initial Electrocardiography showed a high lateral ST elevation myocardial infarction. Although the result of Left cardiac catheterization was normal coronaries, the transthoracic echocardiography (TTE) indicated the presence of severe reduction in the left ventricular function with TTE findings of reverse Takotsubo syndrome. The patient was managed with inotropic support, diuresis, and intra-aortic balloon pump insertion. She showed good clinical improvement, and left ventricular function was recovered within a few days.

**Discussion:**

This case shows that reverse Takotsubo cardiomyopathy may occur in young women, particularly in the postpartum period, undergoing anaesthesia and stress of caesarean section surgery. We recommend awareness of this possible diagnosis and further management of this unexpected variant of acute heart failure. A multidisciplinary approach is recommended to optimize outcomes for the mother and child.

Learning points

- Reverse Takotsubo cardiomyopathy can be a cause of acute heart failure during induction of anaesthesia.
- A multidisciplinary approach is recommended to optimize outcomes for the mother and child.

## Introduction

Takotsubo cardiomyopathy is a type of acute heart failure typically following an emotional or physical stressor. Reverse Takotsubo is a rare pattern representing only 2% of the disease. There are many reported medical triggers, including surgical procedures, intubation, and induction of general anaesthesia.

## Summary figure

**Figure:**
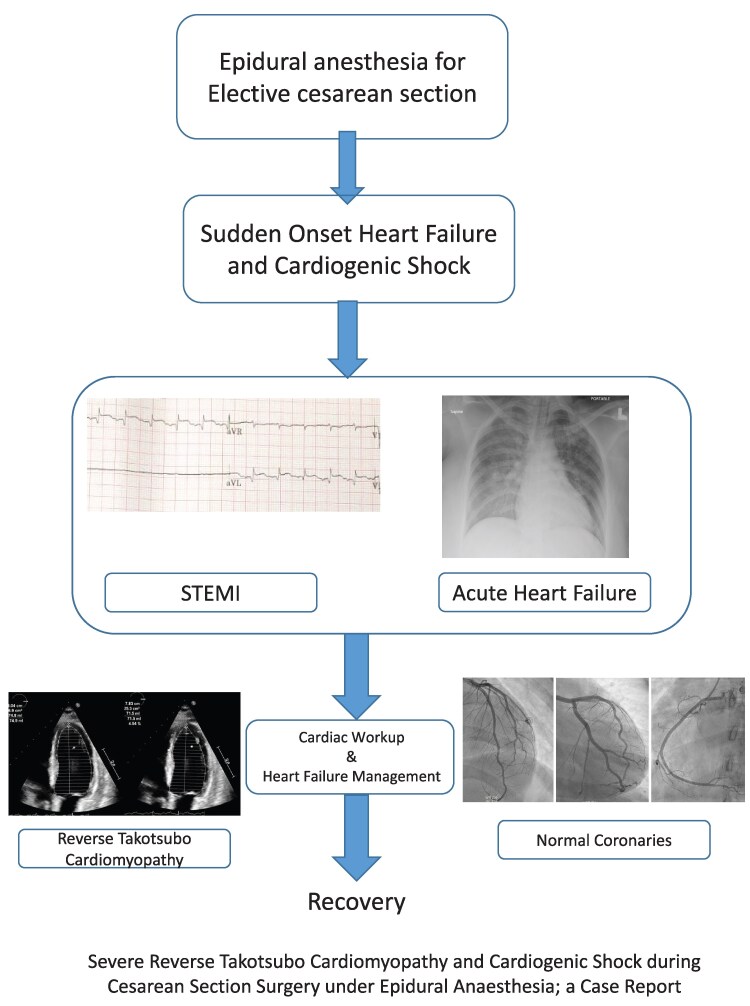


## Case presentation

**Figure 1:**
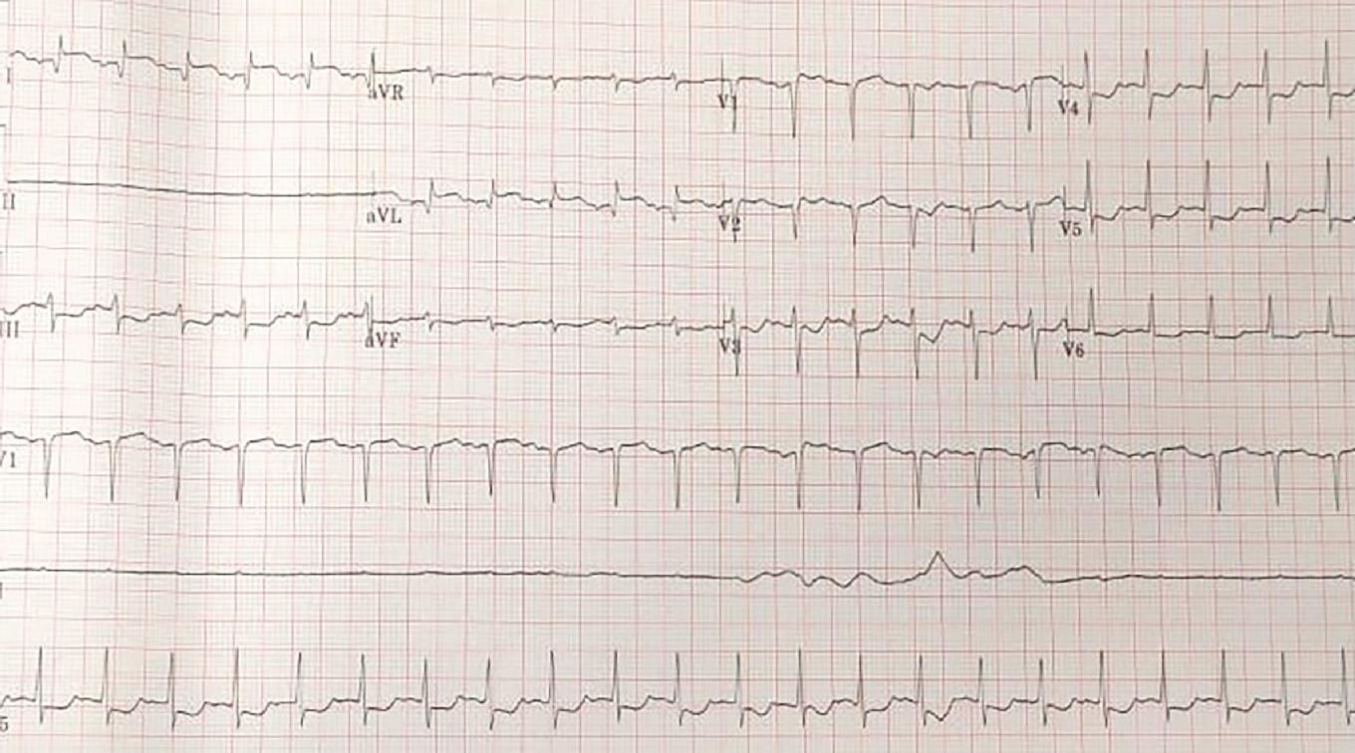
Electrocardiogram showing sinus tachycardia and ST elevation in high lateral leads, I, and aVL, with diffuse ST depression in most of the other leads.

A 27-year-old pregnant female at 39 weeks of gestational age with no past medical history underwent caesarean section surgery under epidural anaesthesia due to failed progression of vaginal delivery. After the induction of epidural anaesthesia and during the surgery, she developed tachypnoea, tachycardia, oxygen desaturation, and hypotension. Hypotension was managed with volume resuscitation and vasopressor medications, and respiration was supported by intubation and mechanical ventilation. Urgently, the caesarean section surgery was completed successfully with the delivery of a single, alive baby girl. The initial electrocardiography (ECG) showed a high lateral ST elevation myocardial infarction (STEMI) (*Figure 1*). Based on that, heparin infusion was started, and the patient was emergently transferred to the tertiary hospital as a case of STEMI with cardiogenic shock for further management and intervention.

The patient’s family denied any significant recent symptoms of chest pain, shortness of breath, lower limb oedema, fever, or any recent infection. Also, there is no family history of cardiac disease or sudden cardiac death. The patient has a history of a previous uncomplicated caesarean section 3 years ago due to failure to descend. The differential diagnosis included peripartum cardiomyopathy, coronary/aortic dissection, acute myocarditis, Takotsubo cardiomyopathy, and acute pulmonary embolism.

**Figure 2:**
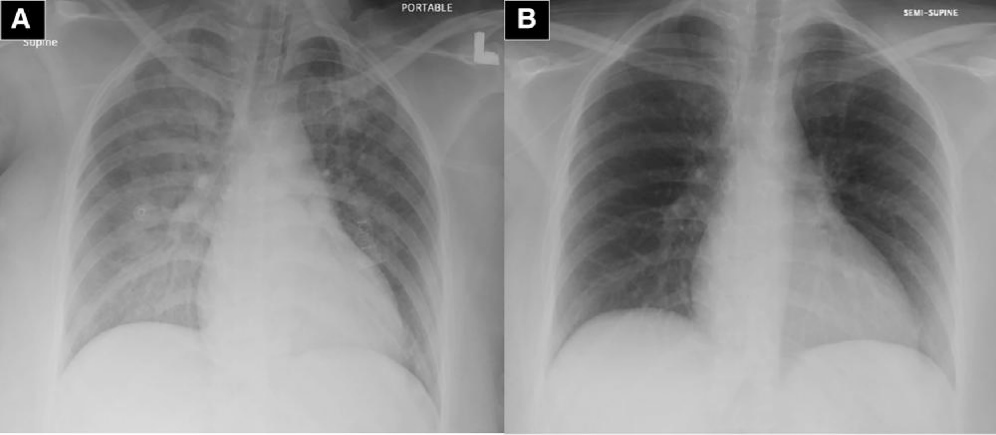
(*A*) Initial chest x-ray on day of admission showing bilateral lung congestion and features of pulmonary oedema. (*B*) A chest x-ray on Day 2 of admission with marked improvement and disappearance of pulmonary congestion.

The initial serum laboratory investigations showed a leucocytosis, white blood cell count (WBC) of 30 × 10^3^/μl (normal 3.9–11 × 10^3^/μl), haematocrit of 37% (normal 37%–52%) and platelet of 345 × 10^3^/μl (normal 150–450 × 10^3^/μl), normal renal function, creatinine of 74 μmol/L (normal 64–104 μmol/L), a significant increase of troponin I level 4486 ng/L (normal <34 ng/L). Chest x-ray (CXR) was congested with signs of pulmonary oedema (*Figure 2A*). Septic screen and nasopharyngeal swab revealed negative viral respiratory pathogens (SARS-CoV-2 virus, influenza A and B virus, H1N1 virus, and MERS-CoV). Additionally, all autoimmune disease screening tests and other infectious viral tests were negative, e.g. hepatitis C virus, hepatitis B virus, human immunodeficiency virus, and Epstein–Barr virus.

**Figure 3:**
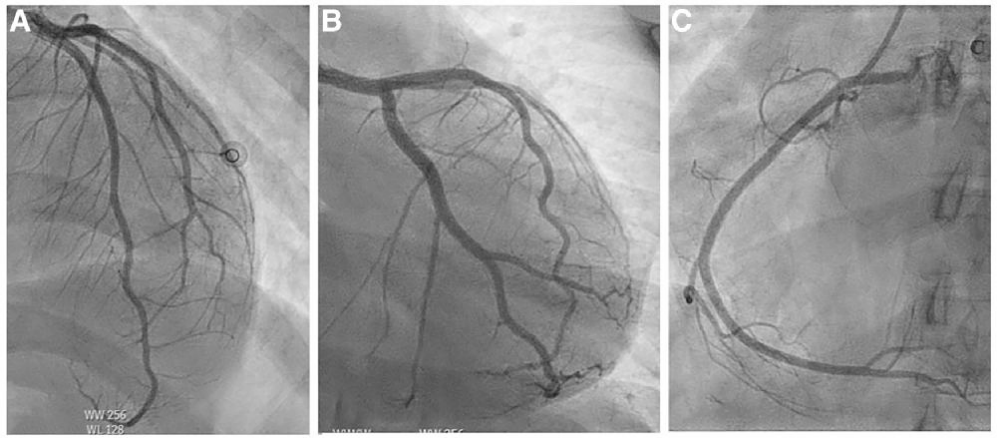
Still images from a coronary angiogram showing normal left anterior descending (*A*), left circumflex artery (*B*), and right coronary artery (*C*).

Transthoracic echocardiography (TTE) demonstrated left ventricular ejection fraction (LVEF) of 30% and a mildly dilated left ventricle (LV) with severe hypokinesia of all basal and mid-segments, with hypercontractility of the apex. These findings were suggestive of a reverse Takotsubo syndrome (see Supplementary material online, *Videos S1*, *S2*, *S3*, *S4*). Left cardiac catheterization showed normal coronaries with left ventricular end-diastolic pressure of 34 mmHg (*Figure 3*).

**Figure 4:**
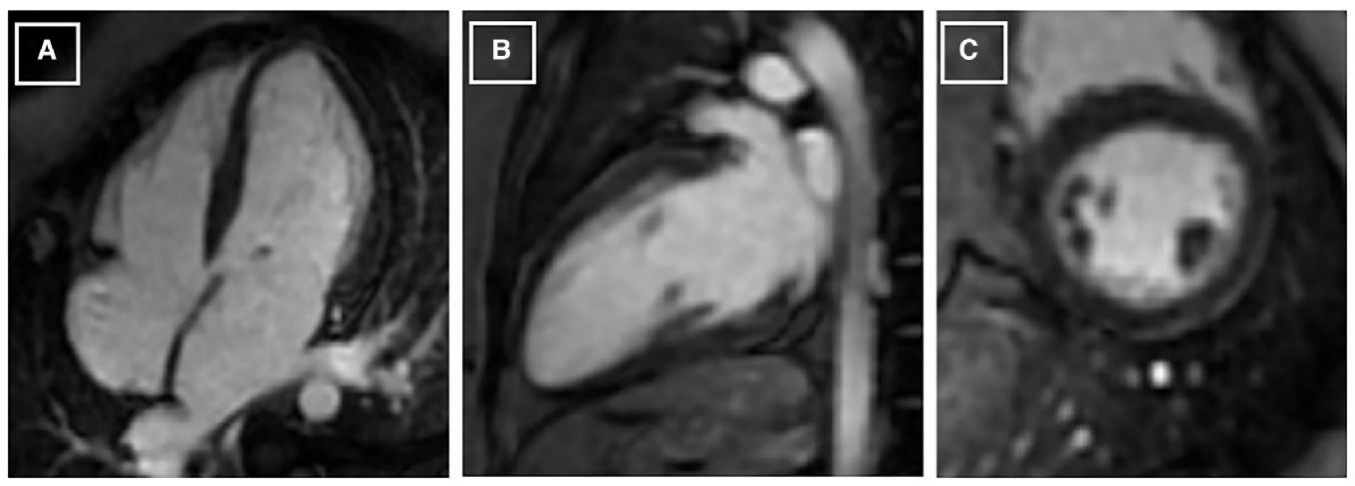
Cardiac magnetic resonance imaging early after contrast (gadolinium) showing no enhancement (Panel *A*, *B* and *C*).

Hypotension was managed by inotropic support and an intra-aortic balloon pump (IABP) to maintain a mean arterial pressure of >65 mmHg. After 24 h of management, the patient showed good clinical improvement with improved blood pressure and good urine output of >150 ml/h. The vasopressors were gradually tapered off, her CXR (*Figure 2B*) improved, and the mechanical ventilation requirement became minimal. On the third day of admission, the patient was extubated, then IABP was removed successfully, leucocytosis improved to near normal (WBC of 12 000 cells/pl), and troponin I level decreased to 439 ng/L (previously 4486 ng/L). The TTE was repeated on the fourth day of admission and showed recovered LVEF to 55% (see Supplementary material online, *Video S5, S6, S7*). Cardiac magnetic resonance was conducted on the fifth day of admission, revealing recovered LV function with no evidence of myocarditis or infiltrative cardiomyopathy (*Figure 4*). Later on, the patient was discharged home on heart failure guideline-directed medical therapy and outpatient cardiology clinic follow-up.

## Discussion

Takotsubo cardiomyopathy, also called stress cardiomyopathy, is a condition in which LV dilatation and acute systolic heart failure occur, typically following emotional or physical stress.^1^ It is characterized by a transient reversible regional hypokinesia of the ventricular myocardium, usually extending beyond the territory perfused by a single coronary artery.^2^ The clinical hallmark of Takotsubo cardiomyopathy is a presentation similar to acute coronary syndrome, with no evident obstructive coronary artery disease.^3^ However, the pathophysiology of Takotsubo cardiomyopathy is complex, and multiple mechanisms have been proposed with no proven conclusive theory.^3,4^ Catecholamine surge is thought to be the leading cause of multivessel spasm, which can affect the coronary macrovascular, microvascular, and peripheral circulations, resulting in cardiac muscle dysfunction.^4^ The classic TTE pattern of Takotsubo cardiomyopathy, also called apical ballooning, is characterized by akinesia of the apex with hypercontractility of the LV base, which almost represents 80% of the cases.^2,5^ On the other hand, reverse Takotsubo is characterized by basal hypokinesis, with normal function of the LV apex, which is a rare pattern representing only 2% of the disease.^5^

The reverse variant differs from the classical pattern and has different clinical features like younger age at presentation, less reduction in LVEF, more frequent ST-T changes in ECG, and lower brain natriuretic peptides levels.^5^ The severity of the disease also differs in this subtype, with less dyspnoea, pulmonary oedema, and cardiogenic shock at presentation.

Peripartum cardiomyopathy and Takotsubo cardiomyopathy during pregnancy are difficult to differentiate, as both are characterized by LV dysfunction with acute heart failure presentation and tend to recover on follow-up.^6,7^ Despite that, patients with Takotsubo cardiomyopathy tend to have a better prognosis, and its management differs from that of peripartum cardiomyopathy, which makes it important to differentiate between both entities.^6–8^ Echocardiography is the initial investigation of choice, and cardiac MRI shows typical regional wall motion abnormalities, with the presence of myocardial oedema and the absence of late gadolinium enhancement as diagnostic hallmarks of the disease.^9^

The management of classical and reverse Takotsubo cardiomyopathy is similar. During acute haemodynamic collapse, the routine inotropes are avoided as they might cause further deterioration of the clinical status, either by increasing the catecholamine surge or by causing LVOT obstruction. Diuretics and vasodilators are also avoided in the obstruction of the outflow, and intravenous fluid, along with early utility of mechanical circulatory support such as a left ventricular assist device, is crucial and life-saving.^10^

In the present case, our patient had two possible factors that can induce acute cardiomyopathy, which are peripartum and Takotsubo cardiomyopathy. Indeed, the circumstances under which the symptoms started suddenly during the caesarean section surgery and epidural anaesthesia, characteristic findings of imaging tests, and the rapid LV recovery within a few days make a diagnosis of Takotsubo cardiomyopathy more likely.

In terms of the possible trigger of Takotsubo cardiomyopathy, the transient LV dysfunction had been reported at the time of the surgical procedure, during intubation and induction of general or spinal anaesthesia, with no clear relationship between a specific anaesthetic medication and Takotsubo cardiomyopathy.^8,11,12^ However, Takotsubo cardiomyopathy is more common in elderly women, and there are very few reported cases of patients during caesarean section surgery.^13–15^ Takotsubo cardiomyopathy is uncommon in pregnancy and during peripartum, and its reverse variant is not reported in this period.^14^ In the present case, we are reporting what we know; the first case presented with the rare reverse Takotsubo syndrome pattern associated with cardiogenic shock, and the likely inducing factors are caesarean section surgery and epidural anaesthesia.

## Conclusion

This case shows that reverse Takotsubo cardiomyopathy may occur in young women, particularly in the postpartum period, undergoing anaesthesia and stress of caesarean section surgery. We recommend awareness of this possible diagnosis and further management of this unexpected variant of acute heart failure. A multidisciplinary approach is recommended to optimize outcomes for the mother and child.

## Supplementary Material

ytaf619_Supplementary_Data

## Data Availability

The data included in this article will be shared upon reasonable request to the corresponding author.

## Lead author biography

Dr. Ammar Alkadi is a cardiologist and echocardiography consultant. He is trained at King Fahad Medical City, Riyadh, Saudi Arabia, and has a special interest in cardiac imaging and research.

## Notes

**Consent:** The authors confirm that written consent for the submission and publication of this case report, including images and associated text, has been obtained from the patient and family in line with COPE guidance.

**Funding:** None.
